# Health Economic Data in Reimbursement of New Medical Technologies: Importance of the Socio-Economic Burden as a Decision-Making Criterion

**DOI:** 10.3389/fphar.2016.00252

**Published:** 2016-08-17

**Authors:** Georgi Iskrov, Svetlan Dermendzhiev, Tsonka Miteva-Katrandzhieva, Rumen Stefanov

**Affiliations:** ^1^Department of Social Medicine and Public Health, Faculty of Public Health, Medical University of PlovdivPlovdiv, Bulgaria; ^2^Institute for Rare DiseasesPlovdiv, Bulgaria; ^3^Section of Occupational Diseases and Toxicology, Second Department of Internal Medicine, Faculty of Medicine, Medical University of PlovdivPlovdiv, Bulgaria

**Keywords:** health technology assessment, reimbursement, decision-making, cost-effectiveness, burden of disease, socio-economic burden, cost-of-illness, rare diseases

## Abstract

**Background:** Assessment and appraisal of new medical technologies require a balance between the interests of different stakeholders. Final decision should take into account the societal value of new therapies.

**Objective:** This perspective paper discusses the socio-economic burden of disease as a specific reimbursement decision-making criterion and calls for the inclusion of it as a counterbalance to the cost-effectiveness and budget impact criteria.

**Results/Conclusions:** Socio-economic burden is a decision-making criterion, accounting for diseases, for which the assessed medical technology is indicated. This indicator is usually researched through cost-of-illness studies that systematically quantify the socio-economic burden of diseases on the individual and on the society. This is a very important consideration as it illustrates direct budgetary consequences of diseases in the health system and indirect costs associated with patient or carer productivity losses. By measuring and comparing the socio-economic burden of different diseases to society, health authorities and payers could benefit in optimizing priority setting and resource allocation. New medical technologies, especially innovative therapies, present an excellent case study for the inclusion of socio-economic burden in reimbursement decision-making. Assessment and appraisal have been greatly concentrated so far on cost-effectiveness and budget impact, marginalizing all other considerations. In this context, data on disease burden and inclusion of explicit criterion of socio-economic burden in reimbursement decision-making may be highly beneficial. Realizing the magnitude of the lost socio-economic contribution resulting from diseases in question could be a reasonable way for policy makers to accept a higher valuation of innovative therapies.

## Introduction

The balance between the value of a health technology and the effective access to it represents an important issue of today’s health policy. Assessment and appraisal of new medical technologies is a debate of political priorities, health system specifics and societal expectations. In all countries, choices in the allocation of resources are necessary. Health technology assessment (HTA) has been introduced as a concept to address rising health care costs and growing fiscal concerns (Iskrov and Stefanov, 2016). Health economic data play a crucial role in this process and the subsequent reimbursement decision-making (Jakovljevic and Getzen, 2016).

Health technology assessment systematically explores the properties and effects of a health technology, evaluating direct, and intended effects, as well as indirect and unintended consequences. These factors include safety, efficacy, effectiveness, cost, cost-effectiveness, as well as expected social, legal, ethical, and political impacts. There is a growing consensus on the importance of balancing all these criteria, which are determining the impact of a health technology on the healthcare system. In this context, the progress in medical research and development requires innovation of HTA process too. HTA should be updated in order to respond to such challenges, as innovative health technologies pose new critical factors, which affect patients, payers and providers (Panzitta et al., 2015).

## Objective

This perspective paper discusses the socio-economic burden of disease as a specific reimbursement decision-making criterion and calls for the inclusion of it as a counterbalance to the cost-effectiveness and budget impact criteria. The current limitations of the latter in HTA of innovative therapies are outlined. We focus on addressing these concerns through cost-of-illness studies. This is illustrated through rare diseases and orphan drugs as a paradigm of innovative health technologies.

## Cost-Effectiveness in Reimbursement Decision-Making

Cost-effectiveness is a leading consideration in priority setting and resource allocation. It is a utilitarianism-inspired idea, aiming to achieve the biggest possible benefits to the widest range of users. Its rationale is clear, as growing number of innovative health technologies are available while budget resources are limited. Cost-effectiveness is usually denoted as an incremental cost-effectiveness ratio (ICER). ICER is defined as the ratio of the change in costs of a therapeutic intervention (compared to the alternative) to the change in effects of the intervention (Eichler et al., 2004). In other words, it is the ratio of the extra costs to the extra effects. Meeting this criterion is the most important objective from health economic perspective. In practice, however, few innovative health technologies tend to be cost-effective. Appraisal is very often a choice between more costs and more effects or less costs and less effects.

Incremental cost-effectiveness ratio is not a new concept. Despite political will and public demand for transparent and objective reimbursement decisions on new medical technologies, there are very few examples of officially accepted and applied ICER thresholds. UK’s National Institute for Health and Care Excellence (NICE) is often mentioned as using, albeit implicitly, ICER thresholds. Nevertheless, this institution has repeatedly denied such statements. ICER does offer a range of theoretical advantages, including reduced burden on decision-makers, consistency and effectiveness of reimbursement decisions (Eichler et al., 2004). However, this criterion remains a politically and morally dividing issue. The implementation of an explicit ICER threshold requires various comparisons and rankings under strictly defined settings, which do not always exist in real world. Moreover, there is no constant, context-independent willingness to pay for each gained unit of health effect (McCabe et al., 2008). Payers and society as a whole tend to give different priority to different health technologies. In many occasions, there is a need for flexibility and inclusion of *ad hoc* considerations in these decisions. Furthermore, the single universal focus on ICER as a reimbursement decision-making benchmark is detrimental. ICER has been criticized for limiting patient choice and health care rationing (Schnipper et al., 2015). Finally, any positive ICER, no matter how appealing, represents additional spending which may not be always affordable or sustainable.

## Budget Impact in Reimbursement Decision-Making

The overall reimbursement decision on a new medical technology requires a budget impact analysis. Opportunity costs are the main reason for implementing this criterion. While various economic analyses allow decision makers to assess the effectiveness of health technologies, budget impact analysis is measuring the financial impact of the adoption and use of a new medical technology within the health system. Given the increasingly stringent budgetary frameworks, regulators and payers demand information on the impact that a new technology would have on their limited budget (Niezen et al., 2009). In other words, this indicator represents an assessment of the accessibility of a new medical technology. Economic analyses provide the basis for a favorable reimbursement decision and budget impact analysis ultimately determines what resources would be needed to actually implement this decision.

It is not surprising that budget impact considerations are sometimes blamed for undermining the rational application of the cost-effectiveness criterion (Niezen et al., 2009). Budget impact is a substantial issue because health authorities attach great importance to the sustainability of the health care system. With regard to new medical technologies, they fear that the costs of these innovative therapies would be significant and may cause changes in resource allocation. In fact, studies showed that health technologies with a high budget impact are much more likely to be rejected for reimbursement or to be subject of access restrictions than technologies with a limited impact (Mauskopf et al., 2013a,b). Furthermore, budget impact analysis is posing some practical challenges for new medical technologies. Especially in the case of highly innovative therapies, data on the size of patient population, secondary costs, degree of market penetration are difficult to estimate. Use of health care information is traditionally fragmented and this is additionally exacerbating the problem. Health care costs are usually divided into several different budgets. Reimbursement decisions are often taken at product level, without considering the spillover effect. For example, an innovative drug may significantly increase the costs for treatment, but at the same time it could also reduce the costs for other health and social services (Iskrov and Stefanov, 2014).

## Extension of the Scope and use of Health Economic Data in Reimbursement Decision-Making

Health economic data and their use could significantly affect subsequent reimbursement decisions (Jakovljevic and Getzen, 2016). It is important that all relevant costs and outcomes of the medical technology in question are identified and measured. Direct and indirect costs should be included. The same goes for positive and negative outcomes. Added societal value must be considered as well. The choice of comparator is crucial and has to be guided only by the evidence-based medicine. When a new medical technology belongs to a well known therapeutic class, this comparison is easily done. Nevertheless, many innovative therapies represent a new therapeutic class themselves. In this case, there is an additional risk for bias in the comparison (Iskrov and Stefanov, 2014).

**FIGURE 1:**
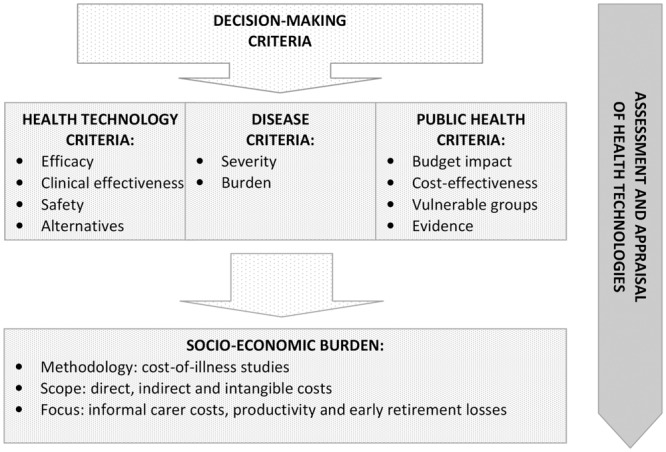
**Socio-economic burden as a decision-making criterion in health technology assessment and appraisal**.

Reimbursement decision-making is not only about cost-effectiveness and budget impact (**Figure 1**). Assessment and appraisal of new medical technologies require a balance between the interests of different stakeholders. Final decision should take into account the societal value of new therapies. HTA itself does not determine whether a new medical technology worth spending of public funds for its use. This decision is ultimately taken by health authorities and payers, who base their recommendations on a combination of other criteria as well, including political factors (Iskrov and Stefanov, 2016). Reimbursement decision-making is always a question of trade-off. In the case of new medical technologies, this issue is even controversial, as it consists of two opposing principles – beneficence and justice. This is why the role of health economic data in reimbursement decision-making should further expanded. A clearly defined and accepted use of socio-economic burden as a reimbursement criterion could counterbalance the domination of the cost-effectiveness and budget impact criteria.

## Socio-Economic Burden

Socio-economic burden is a decision-making criterion, accounting for diseases, for which the assessed medical technology is indicated (**Figure 1**). This measure is usually researched through cost-of-illness studies that systematically quantify the socio-economic burden of diseases on the individual and on the society. This is a very important decision-making consideration as it illustrates direct budgetary consequences of diseases in the health system and indirect costs associated with patient or carer productivity losses (Angelis et al., 2015b). While cost-effectiveness and budget impact are describing the health technology and its application, the socio-economic burden is characterizing the disorder. It is a crucial point in terms of unmet health needs and health inequalities. Accurate knowledge about socio-economic burden is essential to formulate and prioritize health care policies and technologies, as well as to allocate health care resources in accordance with budget constraints in order to achieve health policy efficiency (Jo, 2014).

There is no uniform definition for the burden of disease. High socio-economic burden of disease does not necessarily mean acute condition or frequent hospitalization. This indicator and its values are more related to the degree, by which physical and social symptoms affect the ability of patients to lead a normal life and perform daily activities. It incorporates a high dependence on family, relatives and carers, as well as frequent follow-up by expert medical professionals. Despite being health economic by nature, it is very important to engage clinicians when defining this criterion and its scope (Jakovljevic et al., 2016). Knowledge on disease epidemiology, morbidity and prognosis is crucial. To understand socio-economic burden and to use it efficiently in reimbursement decision-making, it is important to analyze how socio-economic costs are defined, classified and measured. Traditional paradigm of cost-of-illness studies puts costs into three categories: direct, indirect and intangible costs. Nevertheless, the decision-making focus is greatly on the first two groups, as intangible costs are difficult to quantify. Furthermore, the criterion of socio-economic burden of disease represents the potential benefits of a new medical technology if it had eradicated the disease. This type of health economic research is closely related with the concept of disability-adjusted life years, which encompass health care costs, as well as lost socio-economic contribution resulting from premature death or disability (Jo, 2014).

## Socio-Economic Burden of Diseases and Health Technology Assessment

New medical technologies, especially innovative therapies, present an excellent case study for the inclusion of socio-economic burden in HTA and reimbursement decision-making. Innovative therapies are usually seen as recently introduced or modified health technologies with unproven effect or side effect undertaken in the best interest of the patient. They could be anything from an innovation with no precedent to using a conventional treatment in a different context. Assessment and appraisal of these therapies tend to be one of the most complicated tasks for health authorities and payers. It has been acknowledged that, while regulatory incentives have stimulated research and development of innovative therapies on a global level, equitable and timely access to market approved ones remains an issue. HTA has been heavily promoted a health policy tool to ensure sustainability and credibility of the reimbursement decision-making process. Despite best efforts, there are legitimate concerns among medical professionals, patients and industry that access to innovative therapies is greatly delayed (Iskrov and Stefanov, 2014). It should not be forgotten that HTA is only an instrument. Assessment and appraisal of new medical technologies have been greatly concentrated so far on cost-effectiveness and budget impact, marginalizing all other considerations. It is important to underline that reimbursement-decision making is perceived as fair and legitimate, when this process leads to a balance and agreement among different stakeholders’ interests. Reimbursement policy must recognize public health priorities and fiscal constraints, but it should also respect the individual health care right of each patient (Iskrov and Stefanov, 2016).

These assumptions are particularly strong in the field of rare diseases and orphan drugs. Rare diseases pose a unique challenge to health authorities and payers, as they represent life-threatening or chronically debilitating conditions with a low prevalence and a high level of complexity. It is estimated that between 5 000 and 8 000 distinct rare diseases exist today, affecting between 6 and 8% of the population in the course of their lives. In other words, the total number of people affected by rare diseases in the EU is between 27 and 36 million. Because of their low prevalence, their specificity and the high total number of people affected, rare diseases call for a global approach based on special and combined efforts to prevent significant morbidity or avoidable premature mortality and to improve the quality of life and socio-economic potential of affected persons (European Union, 2009).

Methods for health economic evaluation have their own specifics when it comes to rare disease-related orphan medicinal products. Orphan drugs are unable to meet the standard ICER threshold. Moreover, health authorities and payers have strong concerns about the increasing budget impact of those therapies. These two considerations have historically had a negative impact on the assessment and appraisal of orphan drugs (Rosenberg-Yunger et al., 2011; Iskrov and Stefanov, 2014). In this context, data on rare disease burden and inclusion of explicit criterion of socio-economic burden in reimbursement decision-making may be highly beneficial. Realizing the magnitude of the lost socio-economic contribution resulting from rare disease premature death or disability could be a reasonable way for policy makers to accept a higher valuation of innovative therapies. Generation of such evidence is crucial for the timely access to these products.

The EU-funded BURQOL-RD project (Social Economic Burden and Health-Related Quality of Life in Patients with Rare Diseases in Europe) should be highlighted as the very successful first step toward this objective. BURQOL-RD studied both direct and indirect costs for 10 rare diseases in 8 EU Member States. While there were important differences between countries depending on the degree of development of formal care provided by social services, informal care was found to be the main social resource involved in the care of people with rare diseases (López-Bastida et al., 2016). Results from this project showed the importance of studying the economic consequences of rare diseases from a societal perspective and interpreting the outcomes in a global framework. Burden of disease data from BURQOL-RD provided insights into the distribution of rare disease costs and their impact on national health system expenditure, as well as on patient and family income (Angelis et al., 2015a; López-Bastida et al., 2016). More importantly, this study demonstrated that while direct costs for rare diseases were significant, other indirect societal costs, such as informal care, productivity loss and early retirement, were even higher. In short, rare diseases represent considerable invisible costs to the society and this should be taken into account when making a reimbursement decision about orphan drugs and innovative therapies for rare diseases.

## Conclusion

Assessment and appraisal of new medical technologies is a debate of political priorities, health system specifics and societal expectations. Health economic data and their use could significantly affect subsequent reimbursement decisions. Reimbursement decision-making is not only about cost-effectiveness and budget impact. Socio-economic burden quantified through cost-of-illness studies is an important and essential benchmark in health policy. By measuring and comparing the socio-economic burden of different diseases to society, health authorities and payers could benefit in optimizing priority setting and resource allocation.

Generation of such evidence goes far beyond clinical trials and requires multi-stakeholder cooperation and coordination. Early constructive dialog and elaboration of disease-tailored research tools could set the scene for ongoing accumulation of evidence, as well as for proper and timely assessment and appraisal of new medical technologies. Burden of disease data need to be updated to understand the economics of diseases and their changing cost structures. This will enable policymakers to better understand the factors that impact on disease-related expenditure, and will also enable a better-informed distribution of resources.

## Author Contributions

All authors listed, have made substantial, direct and intellectual contribution to the work, and approved it for publication.

## Conflict of Interest Statement

The authors declare that the research was conducted in the absence of any commercial or financial relationships that could be construed as a potential conflict of interest.
